# AngioVac Debulking in Endocarditis Patients with Large, Device-related Vegetations

**DOI:** 10.19102/icrm.2018.090803

**Published:** 2018-08-15

**Authors:** Nikhil Patel, M. Lawson McDonald, Natalie S. Bradford, Justin W. Smith, Elijah H. Beaty, Jason A. Rytlewski, Tony W. Simmons, Patrick Whalen, David X. Zhao, Prashant D. Bhave

**Affiliations:** ^1^Department of Internal Medicine, Wake Forest School of Medicine, Winston-Salem, NC, USA; ^2^Section on Cardiology, Department of Internal Medicine, Wake Forest School of Medicine, Winston-Salem, NC, USA

**Keywords:** AngioVac, device infection, endocarditis

## Abstract

As the number and complexity of cardiovascular implantable electronic devices has increased, so too has the incidence of device-related infections. Such a rise requires that the focus be directed toward developing universal standards for infected lead removal. To date, no consensus currently exists regarding the optimal management of patients with large vegetations (diameter > 2 cm). In these individuals, medical therapy is universally ineffective and they are often too ill for surgical extraction; furthermore, transvenous lead extraction (TLE) carries with it a risk of large septic pulmonary emboli. We present a series of five cases in which the AngioVac thrombectomy system (AngioDynamics Inc., Latham, NY, USA) was used as an adjunct to TLE. Debridement of infected leads prior to percutaneous lead extraction was accomplished as either a bridge to or as concomitant therapy with laser lead removal at our institution. This study included three males and two females with an average age of 52 years. The sizes of vegetations removed from leads ranged from 1.5 cm to 3.9 cm in the largest dimension and the average diameter was 2.65 cm ± 1.1 cm. The vegetations were successfully debulked in all five patients. This suggests that TLE performed with assistance from the AngioVac system (AngioDynamics Inc., Latham, NY, USA) is a safe and effective alternative to open surgical lead removal in patients with large lead vegetations.

## Introduction

Despite improvements in cardiovascular implantable electronic device (CIED) design and functionality, the risk of device infection is on the rise. Between 1997 and 2004, implantation rates for permanent pacemakers (PPMs) and implantable cardioverter-defibrillators (ICDs) increased by 19% and 60%, respectively.^1^ Similarly, the National Hospital Discharge Survey showed that, between 1996 and 2003, the rate of CIED infections increased 3.1-fold (2.8-fold for PPMs and sixfold for ICDs, respectively).^2^ The relationship between device-related infections and the number of implantable devices has been well-studied and consistently demonstrated in recent literature.^3^ Complete removal of the device and its components is recommended following CIED infection; however, the optimal management of large lead vegetations (> 2 cm) remains unclear.^4^ We present a series of five cases in which the AngioVac thrombectomy system (AngioDynamics Inc., Latham, NY, USA) was used to debride large vegetations prior to percutaneous laser lead extraction in order to minimize the risk of septic pulmonary embolism (PE).

## Device description and methods

The AngioVac Venous Drainage Cannula (AngioDynamics Inc., Latham, NY, USA) is a negative-pressure venous drainage cannula that is designed to capture intravascular material from the right-heart circulation through a balloon-actuated, expandable, funnel-shaped distal tip.^5^ Through either percutaneous access or surgical cut-down, the 22-French (Fr) cannula can be advanced over a guidewire under fluoroscopic guidance to access the venous system via the internal jugular or right common femoral veins. Once venous access is gained, the pump circuit—consisting of a percutaneously-placed 17-Fr Bio-Medicus™ reinfusion cannula (Medtronic, Minneapolis, MN, USA) in the right common femoral vein—is connected with the AngioVac device (AngioDynamics Inc., Latham, NY, USA). With the circuit completed, the pump can then be started, allowing for a negative pressure vacuum to evacuate blood and unwanted intravascular material from the ileocaval system and right heart. The removed blood and debris is then passed through the filter, separating out all debris and allowing for the filtered blood to be returned to circulation through the reinfusion cannula in the right common femoral vein.

This procedure is performed by a team of interventional cardiologists and cardiac surgeons, guided by transesophageal electrocardiography (TEE) and intracardiac ultrasound. The procedure requires a single advance of the cannula into the venous system and is indicated for continuous use during extracorporeal bypass for up to six hours. Sufficient debulking of lead vegetations requires an average of 10 minutes divided into continuous bouts of two minutes each. Once sufficient debulking is accomplished, the AngioVac cannula system (AngioDynamics Inc., Latham, NY, USA) is removed and the filter is inspected for thrombi.

## Case presentations

### Case 1

**Figure 1:**
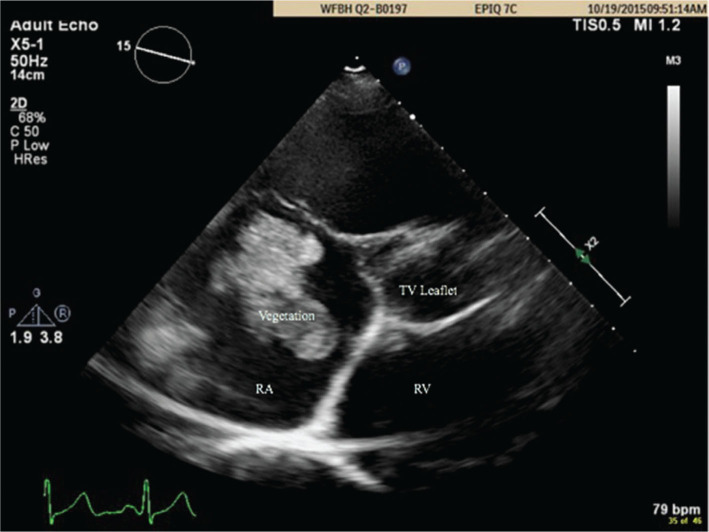
TEE scan demonstrating mobile echo-density in case 1. TV: tricuspid valve; RA: right atrium; RV: right ventricle.

A 46-year-old male with a secondary prevention dual-chamber ICD presented with painless vision loss in his right eye. An evaluation revealed fungal endophthalmitis with blood cultures positive for *Candida albicans*. TEE revealed a 2-cm mobile echodensity attached to the right atrial (RA) lead **(Figure 1)**. Subsequent chest computed tomography results revealed multiple nodules and cavitary lesions consistent with septic emboli. The patient was deemed to be high-risk for complications from open surgical lead removal and the decision was made to use the AngioVac cannula (AngioDynamics Inc., Latham, NY, USA) to debulk the vegetation prior to percutaneous lead removal.

**Figure 2:**
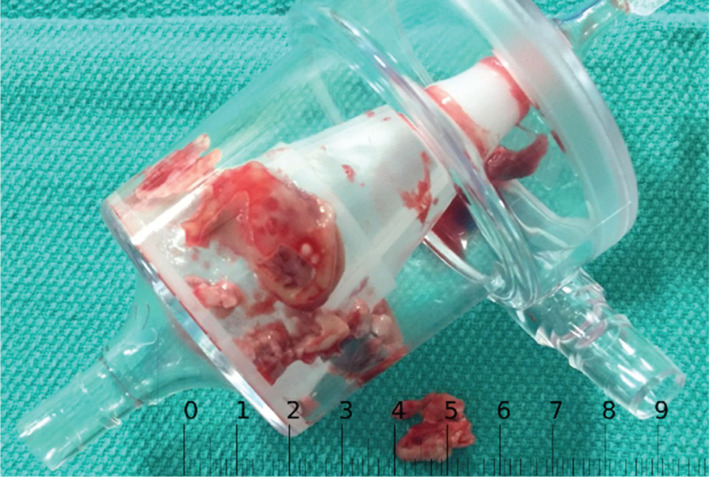
Vegetation material extracted into the AngioVac filter (AngioDynamics Inc., Latham, NY, USA) in case 1.

Debridement of the RA lead was successfully completed and subsequent transvenous extraction of the RA and right ventricular (RV) ICD leads was accomplished without complications. The procedure was performed under general anesthesia using TEE and fluoroscopic guidance. A 24-Fr extracorporeal membrane oxygenation (ECMO) cannula was inserted into the right femoral vein using a modified Seldinger technique with serial dilation; this was subsequently connected to the ECMO pump for use as a reinfusion port. Anticoagulation with unfractionated heparin was used to maintain an activated clotting time of more than 250 seconds. The AngioVac cannula (AngioDynamics Inc., Latham, NY, USA) was then prepped and advanced to the inferior vena cava over a J-wire via the left femoral vein. The pump circuit was primed with warm saline solution, connected to the AngioVac cannula (AngioDynamics Inc., Latham, NY, USA) and reinfusion cannula, and cleared of luminal air. Suction was then initiated. Multiple suction runs covering the RA and RV leads were then made under fluoroscopic and echocardiographic guidance. Significant debulking was achieved with the extraction of a large amount of vegetation material into the filter **(Figure 2)**. The AngioVac device (AngioDynamics Inc., Latham, NY, USA) and the ECMO cannula were subsequently both removed. Z-stitch sutures were used to achieve hemostasis at both sites. Follow-up TEE revealed no signs of residual vegetation. The patient’s ICD system was explanted using a standard transvenous lead extraction technique. Following the procedure, he completed a six-week course of intravenous micafungin and has been maintained on chronic oral suppressive antifungal therapy with no plan in existence at this time for reimplantation.

### Case 2

A 50-year-old male with a history of cardiomyopathy and congestive heart failure with prior implantation of a cardiac resynchronization therapy defibrillator (CRT-D) presented on referral for the management of septic shock secondary to methicillin-susceptible *Staphylococcus aureus* (MSSA). A TEE scan revealed a mobile, elongated echodensity (3.2 cm × 1.3 cm) adherent to the RA ICD lead. Due to a high risk of pulmonary embolization during lead extraction, the patient was scheduled for AngioVac debridement of the vegetation beforehand. Debulking of the vegetation was successfully achieved and all leads were removed. Postextraction TEE revealed no complications from the procedure.

Reimplantation was delayed due to coagulase-negative staph bacteremia of an unknown source, but the infection was ultimately cleared with daptomycin antibiotic therapy and reimplantation was completed four months after lead removal.

### Case 3

A 68-year-old male with atrial fibrillation and a permanent pacemaker presented with septic shock. On presentation, he was found to have pneumonia with significant anemia and thrombocytopenia. Blood cultures were positive for *Haemophilus parainfluenzae.* TEE showed tricuspid and mitral valve vegetations as well as mobile masses on the pacemaker device. The decision was made to remove the pacemaker generator and extract the leads.

Eight days following laser lead extraction of the device, the AngioVac system (AngioDynamics Inc., Latham, NY, USA) was used to debulk remaining vegetative growth. Postprocedural TEE results showed a reduction in the size of the anterior tricuspid leaflet vegetation. The patient remained afebrile after the procedure with a leukocytosis that improved in the following week. This was followed by a 30-day course of ceftriaxone therapy. The patient was successfully reimplanted with a biventricular PPM two months after initial device explant.

### Case 4

A 45-year-old female with heart failure, cardiomyopathy, and a dual-chamber ICD presented with acute-on-chronic respiratory failure secondary to a right lower lobe subsegmental pulmonary embolism. Blood cultures were positive for *Enterobacter faecalis*. Infective endocarditis was confirmed by a TEE scan, which demonstrated a 3.9 cm × 1.3 cm echodensity on the posterior leaflet of the tricuspid valve as well as a vegetation on the ICD lead. The patient was not a candidate for surgery because of her acute illness and multiple comorbidities.

The patient underwent extraction of the vegetation on the posterior tricuspid valve leaflet via AngioVac use prior to the removal of her ICD generator and leads. Significant debulking was achieved, with a large amount of vegetation material extracted into the filter. Following successful debulking, the ICD generator was removed and the leads were extracted percutaneously. Post-extraction, the patient completed a six-week course of intravenous ceftriaxone and ampicillin and elected to forgo reimplantation.

### Case 5

A 68-year-old female with Hodgkin’s lymphoma, breast cancer, and a biventricular PPM placed for complete heart block presented with septic shock and acute respiratory failure. Blood cultures grew MSSA. TEE demonstrated a 1.5 cm × 1.0 cm mobile echodensity on the tricuspid valve with multiple mobile elongated and circular echodensities on the RA and RV leads. The patient was deemed to be a poor surgical candidate due to her tenuous clinical condition. Percutaneous device extraction was planned with AngioVac vegetation debridement prior to laser lead extraction.

The patient underwent the AngioVac procedure and significant debulking was achieved, with a large amount of vegetation material extracted into the filter. The next day, the patient underwent successful transvenous laser extraction of her three pacemaker leads. Over the next 24 hours, the patient deteriorated with worsening renal function, lactic acidosis, and laboratory value changes consistent with disseminated intravascular coagulation (DIC). Following a discussion with family members, the patient was transitioned to comfort care and expired shortly thereafter.

## Results

A total of five patients with cardiac devices who developed endocarditis underwent debulking of large lead-related vegetations with the AngioVac cannula (AngioDynamics Inc., Latham, NY, USA) as an adjunct to percutaneous lead extraction. This study included three males and two females with an average age of 52 years ± 10.7 years. Devices requiring debulking included three ICDs and two PPMs. The size of the vegetations removed from leads prior to explantation ranged from 1.5 cm to 3.9 cm in the largest dimension and the average diameter was 2.65 cm ± 1.1 cm **(Table 1)**. Vegetations were most commonly found on RA leads with accompanying tricuspid valve vegetation. Extraction was successful in all cases. All patients received long-term antibiotic treatment to reduce the risk of recurrent endocarditis **(Table 2)**. Two patients underwent device reimplantation. One patient expired after lead extraction due to DIC. Postoperative TEE results of each patient, with the exception of those in case 5, demonstrated successful debulking of valvular vegetations **(Table 3)**.

## Discussion

**Figure 3:**
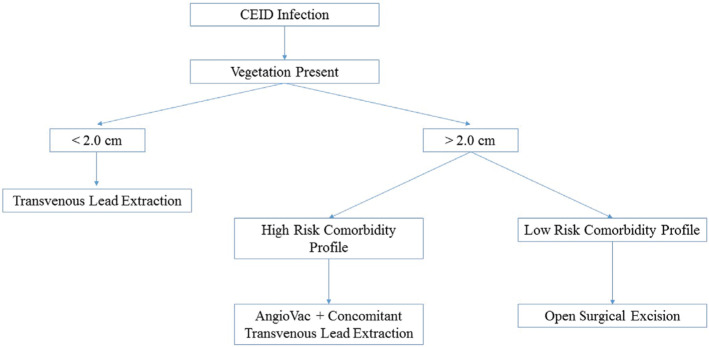
Proposed workflow algorithm for the use of the AngioVac system (AngioDynamics Inc., Latham, NY, USA). CIED: cardiovascular implantable electronic device.

We present five cases in which endovascular lead debulking of large vegetations with the AngioVac cannula (AngioDynamics Inc., Latham, NY, USA) was effective **(Table 1)**. In two of these cases, AngioVac-assisted debridement and laser lead removal were performed as concomitant procedures in a single session. This study adds to the growing body of evidence that endovascular AngioVac debridement of CIED lead vegetation prior to lead removal is a safe and minimally invasive alternative to traditional methods.^6–8^ Previous recommendations call for surgical lead removal by open thoracotomy for vegetations of > 1 cm in diameter.^6^ Open surgical removal is noted for its potential to reduce the risk of septic pulmonary embolism but caries a high risk of severe postoperative complications, including respiratory failure.^9^ Such complications delay patient recovery and increase the cost of postoperative care.^10,11^ Furthermore, patients requiring CIED removal are often characterized by numerous concomitant chronic conditions such as congestive heart failure, poor baseline lung function, and a history of recurrent deep vein thrombosis/PE, making them poor candidates for surgery. In contrast, the AngioVac cannula (AngioDynamics Inc., Latham, NY, USA) offers a less invasive approach to vegetation debridement that both reduces the risk of septic pulmonary embolism and also minimizes the pulmonary complications associated with open surgical lead removal. We have included a proposed workflow algorithm for the use of the AngioVac system (AngioDynamics Inc., Latham, NY, USA) in clinical practice as an alternative to open thoracotomy **(Figure 3)**. To our knowledge, there is only one other case of concomitant AngioVac cannula vegetation debridement and laser lead extraction that has been reported to date,^12^ making the two studies that discuss such in this case series of increased importance. Furthermore, as the periprocedural death that we report in case 5 occurred in a critically ill patient with no feasible alternatives for treatment and was not due to AngioVac use, our findings support the safety and efficacy of the AngioVac cannula (AngioDynamics Inc., Latham, NY, USA) for debulking of large lead and tricuspid valve vegetations prior to laser lead extraction. We also suggest the potential for the concomitant application of these procedures as an effective strategy for reducing the intraoperative risk of septic PE during lead extraction.

## Figures and Tables

**Table 1:** A Summary of Patients Who Underwent Debulking via AngioVac Cannula Use

Case	Description of Patient	Specs	Vegetation Size	Vegetation Location	Device(s) Removed	Why Diagnosed?	Organism
1	46-year-old Caucasian male with AF, COPD, and HTN	ICD with two leads	2 cm × 2 cm grapelike cluster; mobile echodensity consistent with vegetative growth	RA	ICD generator and RA and RV leads explanted	Patient presented with worsening vision in right eye *sans* pain and underwent an examination that revealed fungal endophthalmitis, which further revealed fungal endocarditis	*C. ablicans*
2	50-year-old Caucasian male with cardiomyopathy and CHF	CRT-D with three leads	3.2 cm × 1.3 cm; smaller vegetation on RA roof	RA	CRT-D generator and RA, RV, and LV leads explanted	Patient presented with fatigue, lethargy, and back pain upon admission, coupled with hypotension and sustained atrial flutter	*E. cloacae*
3	68-year-old Caucasian male with diabetes mellitus, HTN, and AF	PPM with three leads	Not applicable	RA/tricuspid valve/mitral valve	PPM generator and RA, RV, MV/CS, and capped/retained RV leads explanted	Patient presented to the ED with features of SIRS/sepsis secondary to *H. parainfluenza* bacteremia having undergone a dental procedure a month prior to presentation	*H. parainfluenza*
4	45-year-old African-American female with HTN, cardiomyopathy, congestive heart failure, obstructive sleep apnea, and sarcoidosis	ICD with two leads	3.9 cm × 1.3 cm on posterior tricuspid valve leaflet	RA/tricuspid valve	ICD generator and RA and RV leads explanted	Patient presented to the ED with shortness of breath and pleuritic chest pain and was found to have acute-on-chronic respiratory failure secondary to *E. faecalis* bacteremia	*E. faecalis*
5	68-year-old Caucasian female with Hodgkin’s lymphoma, right-sided breast cancer, complete heart block, and AF	PPM with two leads	1.5 cm × 1.0 cm on tricuspid valve leaflet	Tricuspid valve	PPM generator and RA, RV, and LV leads explanted	Patient presented to the ED with sepsis, leukocytosis, and fever and was noted to have a draining fistula between her skin and pacemaker cavity	MSSA

AF: atrial fibrillation; COPD: chronic obstructive pulmonary disease; HTN: hypertension; ICD: implantable cardioverter-defibrillator; RA: right atrial/atrium; RV: right ventricle; CHF: congestive heart failure; CRT-D: cardiac resynchronization therapy-defibrillation; LV: left ventricle; PPM: permanent pacemaker; MV/CS: mitral valve/coronary sinus; ED: emergency department; SIRS: systemic inflammatory response syndrome; ICD: implantable cardioverter-defibrillator; MSSA: methicillin-susceptible *Staphylococcus aureus*.

**Table 2:** Follow-up Care After AngioVac Cannula Debulking

Case	Treatment	
1	•	Six weeks intravenous micafungin with transition to oral 400 mg daily fluconazole for lifelong suppression (with the potential to decrease to an oral 200 mg dose)
	•	Concomitant intravenous voriconazole for eye treatment
2	•	Follow-up with nafcillin and ertapenam
	•	Daptomyocin started for bacteremia of unknown etiology
3	•	Follow-up with four weeks of ceftriaxone/unasyn
	•	Four-week unasyn regimen changed to an additional three-week augmentin regimen due to lung abscess
4	•	Intravenous ceftriaxone and ampicillin for six weeks
5	•	Patient deceased

**Table 3:** Follow-up TEE Imaging Results

Case	Post-Procedural TEE	
1	•	Tricuspid valve vegetations were absent
2	•	The right atrium was mildly dilated with absent atrial roof vegetations
3	•	Tricuspid valve vegetations were absent
	•	Mitral valve vegetation absent on subsequent TEE scan
4	•	Tricuspid valve vegetations were absent
5	•	Patient deceased

TEE: transesophageal electrocardiography.
